# Global trends in recombinant human growth hormone for the treatment of idiopathic short stature: a bibliometric analysis

**DOI:** 10.3389/fmed.2025.1577396

**Published:** 2025-08-06

**Authors:** Rong Ouyang, Haiyun Gu, Jie Yuan, Yi Sun, Xiaoqin Zhao

**Affiliations:** Department of Endocrinology, Affiliated Hospital of Nantong University, Nantong, Jiangsu, China

**Keywords:** recombinant human growth hormone, idiopathic short stature, bibliometric, BIB, global trends

## Abstract

**Background:**

The treatment of idiopathic short stature (ISS) with recombinant human growth hormone (rhGH) has been a subject of extensive research. This study aims to perform a bibliometric analysis of publications related to rhGH treatment for ISS, identifying research hotspots, key publications, and international collaboration networks.

**Methods:**

A literature search was conducted on the Web of Science Core Collection, covering literature from 1991 to 2024. Bibliometric tools including CiteSpace, VOSviewer and “bibliometrix” package of R were used to analyze publication trends, authorship, institutional contributions, and citation networks. Keyword co-occurrence and burst detection were performed to identify emerging research topics.

**Results:**

This area of study had experienced significant growth and maturation over the past three decades, characterized by increasing interest and investment in research pertaining to rhGH interventions for ISS. The majority of research output was concentrated in China. Leading contributors to this body of work included the University of Ulsan. The most prolific academic journals in this field were the *Journal of Clinical Endocrinology & Metabolism*. The keyword co-occurrence analysis identified “gene,” “mutations,” and “genotype,” highlighting genetic factors in rhGH therapy for ISS. Keyword burst analysis, however, emphasized recent trends like “safety” and “growth hormone deficiency,” reflecting growing attention to treatment risks and patient-specific care.

**Conclusion:**

This bibliometric analysis highlights the progression of rhGH research for ISS, shifting from foundational studies to contemporary priorities such as tailored therapies and clinical outcomes. Future research should focus on advancing precision medicine and optimizing treatment protocols while addressing safety concerns and long-term effectiveness.

## Introduction

Idiopathic short stature (ISS) is defined as a condition where children’s height is more than two standard deviations below the mean for their age, sex and population group, without any identifiable systemic, endocrine, nutritional, or chromosome-related disorders (1, 2). Affecting an estimated 2.5% of the general population, ISS remains a challenging diagnosis due to its multifactorial etiology, which includes both genetic and environmental determinants (3, 4). Although ISS is typically considered a benign condition, the psychosocial impact on affected children can be profound. Children with ISS often experience lowered self-esteem, social isolation, and diminished quality of life due to their short stature, which can lead to long-term emotional and psychological consequences (5). These challenges underscore the importance of finding effective treatment modalities.

Recombinant human growth hormone (rhGH) has emerged as a significant therapeutic intervention for managing short stature, including ISS (6). Initially developed for growth hormone deficiency (GHD), rhGH was later approved by the U.S. Food and Drug Administration (FDA) in 2003 for treating ISS in children whose height standard deviation scores are ≤−2.25 and whose growth rates suggest they will not reach an adult height within their normal range (7). The approval was based on a series of clinical trials demonstrating that rhGH can increase growth velocity and improve final adult height in children with ISS, though the magnitude of improvement can vary based on numerous factors, including dose, duration, and individual variability (8).

Despite these advancements, the use of rhGH for ISS remains a subject of ongoing debate. While some studies have shown that rhGH can effectively increase height, concerns persist regarding its long-term safety, cost-effectiveness, and the psychosocial benefits relative to its high financial costs (9). Additionally, there is ongoing controversy surrounding the ethical implications of using growth hormone for non-deficiency-related short stature. Some clinicians and ethicists question whether short stature alone constitutes a medical condition that warrants pharmacological intervention, particularly when the benefits are modest and the risks, such as potential metabolic disturbances, are still under investigation (10). Moreover, the criteria for determining which children qualify for rhGH therapy vary across different countries and clinical guidelines, further complicating the issue (11).

Given the growing body of literature on rhGH treatment for ISS, bibliometric analysis offers a powerful method for systematically reviewing and mapping this research landscape (8). Bibliometric analysis is a quantitative approach that examines academic literature to identify research trends, significant publications, and collaborative networks within a specific field (12, 13). This method is particularly useful in capturing the evolution of research topics, highlighting key contributors, and revealing emerging areas of interest. Through analysis, bibliometric studies can assess the impact of individual publications and authors, providing insights into which studies have shaped clinical practice and policy decisions (14, 15). Furthermore, bibliometric tools such as CiteSpace, VOSviewer, and R enable the visualization of co-authorship networks and keyword co-occurrence, which can illustrate the interconnectedness of research groups and institutions (16).

In the context of ISS and rhGH therapy, bibliometric analysis serves several important purposes. First, it allows researchers and clinicians to identify the most influential studies that have shaped the current understanding of ISS treatment. Second, it highlights gaps in the literature where further research is needed, such as long-term safety outcomes or the personalization of rhGH therapy based on genetic predictors (17). Third, it enables the identification of emerging research trends, such as the increasing focus on individualized treatment strategies and value-based care, aligning with broader trends in precision medicine (18). Finally, bibliometric analysis can reveal patterns of international collaboration, offering insights into how research on ISS and rhGH is distributed globally and which countries or institutions are leading the field.

To date, few comprehensive bibliometric analyses have focused specifically on rhGH treatment for ISS, despite the increasing number of publications in this area. Therefore, this study aims to fill that gap by conducting a systematic and comprehensive bibliometric analysis of the literature on rhGH therapy for ISS. By analyzing publication trends, citation patterns, and collaborative networks, this study seeks to identify critical contributions to the field and map out emerging research paradigms.

## Materials and methods

### Search strategy and data collection

A systematic literature search was performed in the Web of Science Core Collection (WoSCC) database, covering publications from January 1, 1991 to June 17, 2024. The search query was: TS = (“recombinant human growth hormone” OR “rh-GH” OR “Humatrope” OR “Human Somatotrophin” OR “Recombinant Somtropin Humatrop” OR “Recombinant Human Somatropin” OR “Maxomat” OR “Growtropin I1” OR “DNA-rhGH” OR “BioHGH” OR “Umatrope”) AND TS = (“Idiopathic short stature” OR “ISS”).

The search was restricted to English-language articles. All retrieved records were exported in plain text format, including full bibliographic information and cited references. The following variables were extracted from each article: publication count, citation count, title, authorship (including first author), institutional affiliation (full name when available), and country/region of origin.

### Statistical analysis

A comprehensive bibliometric analysis was conducted using three primary tools: VOSviewer (version 1.6.20) for constructing and visualizing collaboration, co-citation, and keyword co-occurrence networks (16). In these network maps, node size and color indicate the number and type of items, while the thickness of connecting lines represents the strength of collaborations or co-citations. CiteSpace (version 6.3. R1) for detecting emerging trends and citation bursts in keywords (19). R package “Bibliometrix” (version4.3.3) for descriptive (performance) analysis and data export (12, 20).

Bibliometric indicators, including the H-index (quantifying academic impact), G-index (emphasizing highly cited publications), and M-index (H-index divided by years since first publication), were calculated to assess author and journal influence (21–23). H-index values for each author were obtained from WoSCC data. The impact factor (IF) of journals was extracted from the 2023 Journal Citation Reports (JCR) (24). Microsoft Excel was used for additional calculations and tabulation of bibliometric indicators. A summary of the statistical methods and bibliometric indicators used in this study is provided in Supplementary Table S1.

## Results

### Overview of the main information

**Figure 1:**
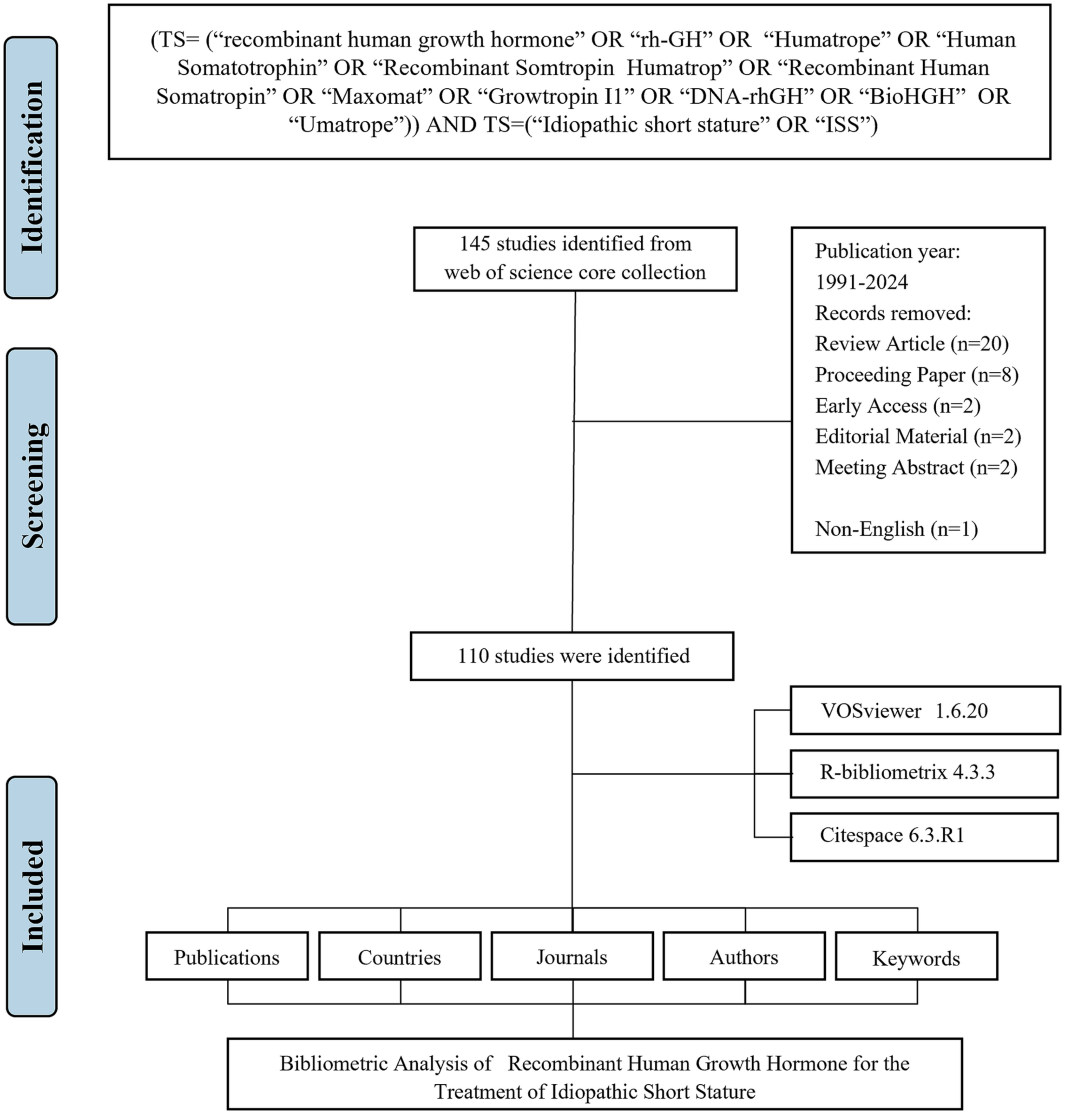
Flowchart of the literature screening process.

**Figure 2:**
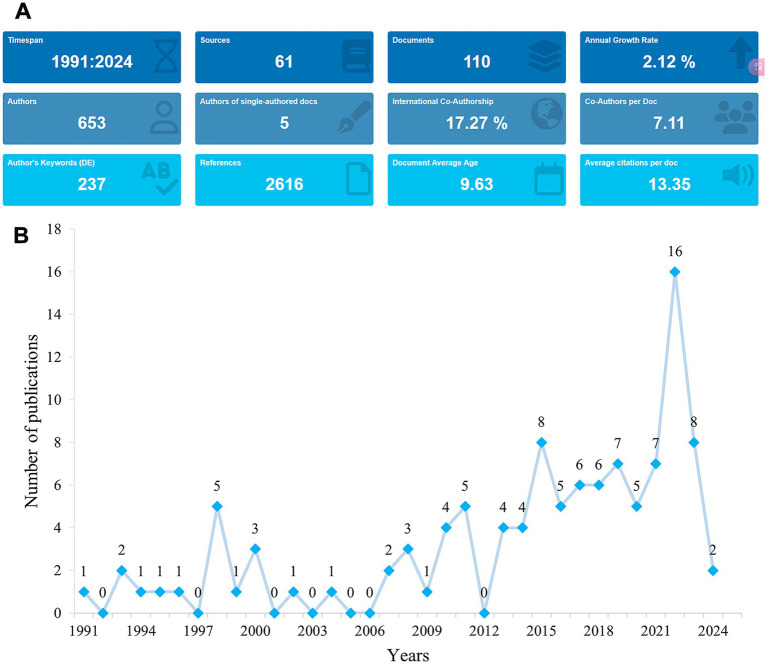
Trends in publications in rhGH treatment in ISS from 1991 to 2024. **(A)** Graphical overview of the total publications per year. **(B)** Detailed trend analysis showing the annual fluctuations in publication volume.

The flowchart of the data screening process is shown in Figure 1. Between 1991 and 2024, a total of 110 articles related to rhGH therapy for ISS were identified for bibliometric analysis (Figure 2A). The publication trend showed a fluctuating yet generally steady increase in research output over the years, with a distinct surge in recent years (Figure 2B). The cumulative growth curve, particularly after 2015, demonstrated a marked increase, indicating intensified research focus in this area. Notably, 2022 recorded the highest number of publications.

### Analysis of journals

The 110 publications were distributed across 35 journals, with a higher number appearing in journals specializing in pediatric endocrinology and growth disorders, which commonly publish research on rhGH therapy for ISS (Supplementary Table S1). The journal with the highest total citations was the *Journal of Clinical Endocrinology & Metabolism* (TC = 623), with 8 publications, making it the most impactful journal in this field. This was followed by *Hormone Research in Paediatrics*, which had 11 publications and ranked second in total citations (TC = 144). *Journal of Pediatric Endocrinology & Metabolism* ranked second in terms of total publications (TP = 10) but had a lower citation count (TC = 82). The journal with the highest impact factor (IF) was *Analytical Chemistry* (IF = 6.7).

**Figure 3:**
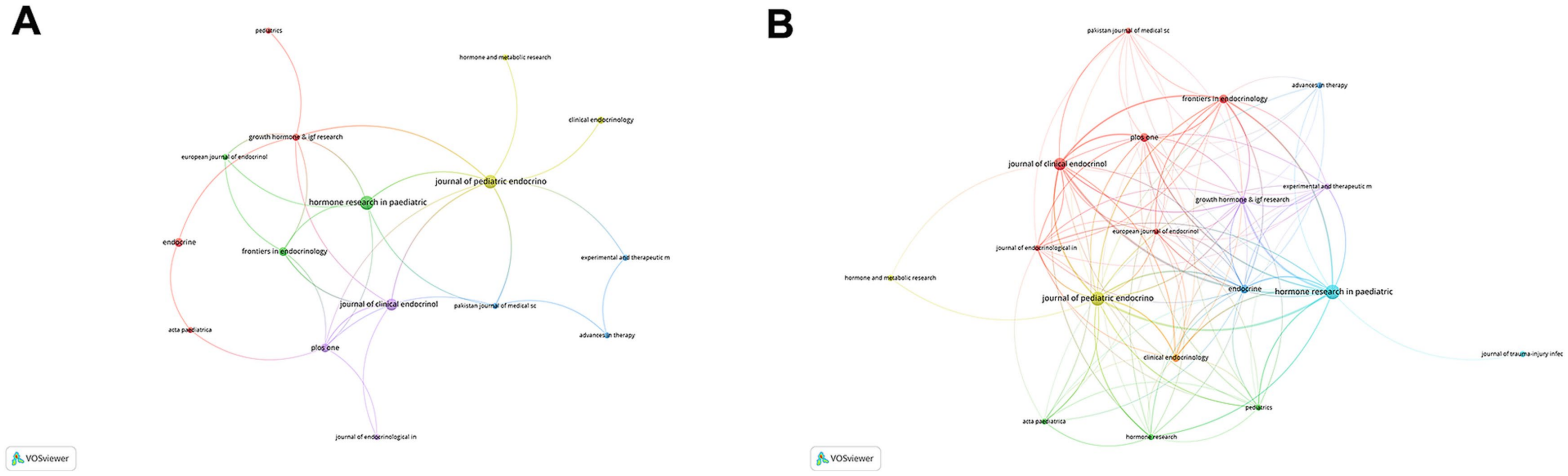
Network analysis of journals in rhGH treatment in ISS. **(A)** Co-occurrence Networks of Journals, reflecting thematic or topical connections based on co-citations. **(B)** Coupling Networks of Journals, illustrating shared intellectual foundations through common references.

The Co-occurrence Networks of Journals (Figure 3A) map thematic or topical connections between journals based on co-citations. The visual network reveals that Hormone Research in Paediatrics, Journal of Pediatric Endocrinology & Metabolism, and Journal of Clinical Endocrinology & Metabolism are central nodes, indicating their significant contributions to the field. These journals are closely connected to other prominent publications such as Frontiers in Endocrinology and Pediatrics. The Coupling Networks of Journals (Figure 3B) highlight shared intellectual foundations through common references. This network illustrates dense connections among highly cited journals, further underscoring the foundational role of these publications in shaping research on rhGH treatment. Notably, Hormone Research in Paediatrics and Journal of Clinical Endocrinology & Metabolism form strong links with various other endocrinology and pediatric journals, indicating their pivotal role in advancing both clinical practice and research theory in this field.

### Country/region analysis

**Figure 4:**
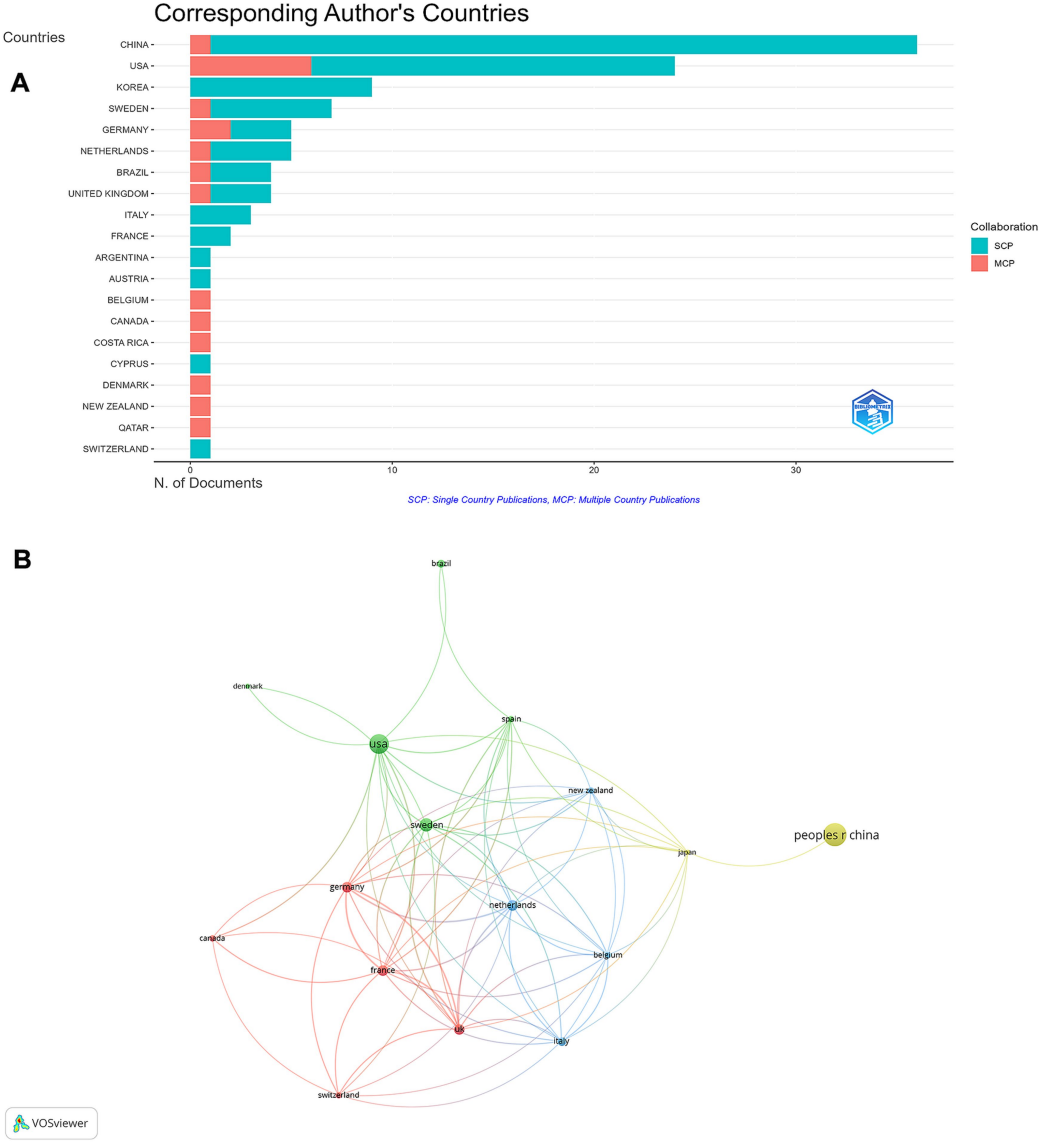
Global distribution and collaboration in rhGH treatment in ISS. **(A)** Corresponding authors’ publications by country, differentiating between single country publications (SCP) and multiple country publications (MCP). **(B)** Collaboration map among countries.

On a global scale, research on rhGH treatment for ISS spanned the top 20 countries by publication volume, with China (n = 36) leading, followed by the USA (*n* = 24) and South Korea (*n* = 9) (Supplementary Table S2). In terms of multiple country publications (MCP), the United States and Germany had higher outputs, with MCP = 6 and 2, respectively. In contrast, China produced more publications as a single country (SCP = 35) (Figure 4A). The international collaboration network analysis revealed that China had the highest total link strength, indicating the strongest international collaborations, followed by the USA, Germany, and Sweden (Figure 4B). Despite China’s leadership in both publication output and collaboration strength, there remains potential to expand partnerships with other countries to further enhance global research efforts.

### Institution analysis

**Figure 5:**
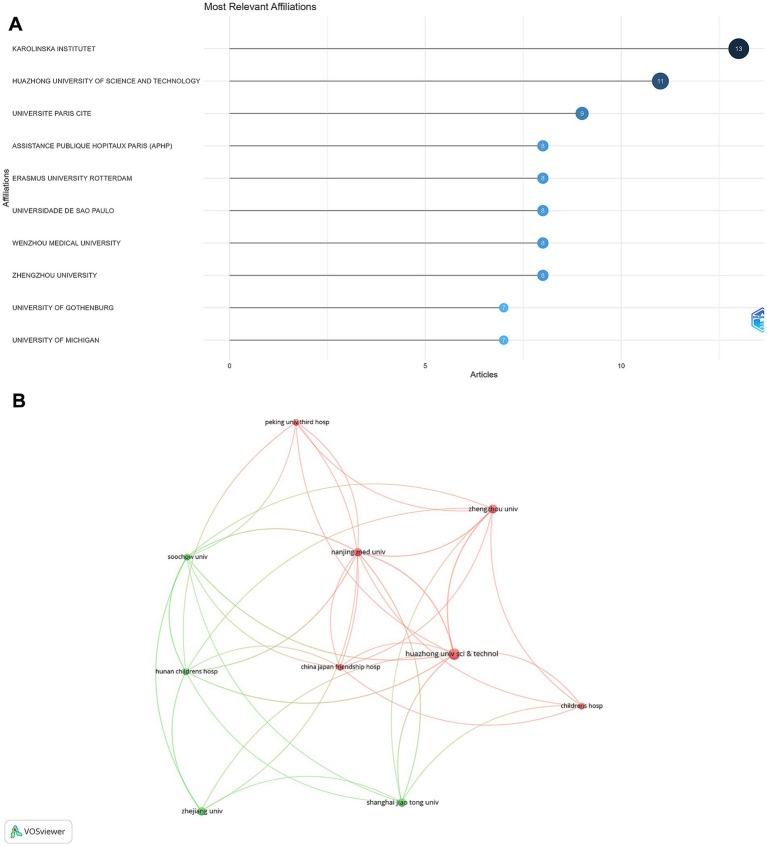
Institutional contributions and collaborations in rhGH treatment in ISS. **(A)** Top 10 institutions by article count and rank. **(B)** Collaboration map among institutions.

The Karolinska Institute in Sweden emerged as the most productive institution, contributing 13 articles (Figure 5A). This was followed by Huazhong University of Science and Technology in China, with 11 articles. Other notable institutions included Université Paris Cité (9 articles); Assistance Publique Hôpitaux de Paris (AP-HP); Erasmus University Rotterdam; Universidade de São Paulo; Wenzhou Medical University; and Zhengzhou University, each contributing 8 articles. The institutional collaboration network analysis revealed strong national clusters (Figure 5B), particularly among South Korean and Chinese institutions. In contrast, international collaborations were less frequent but were observed between certain European and North American institutions.

### Author analysis

**Figure 6:**
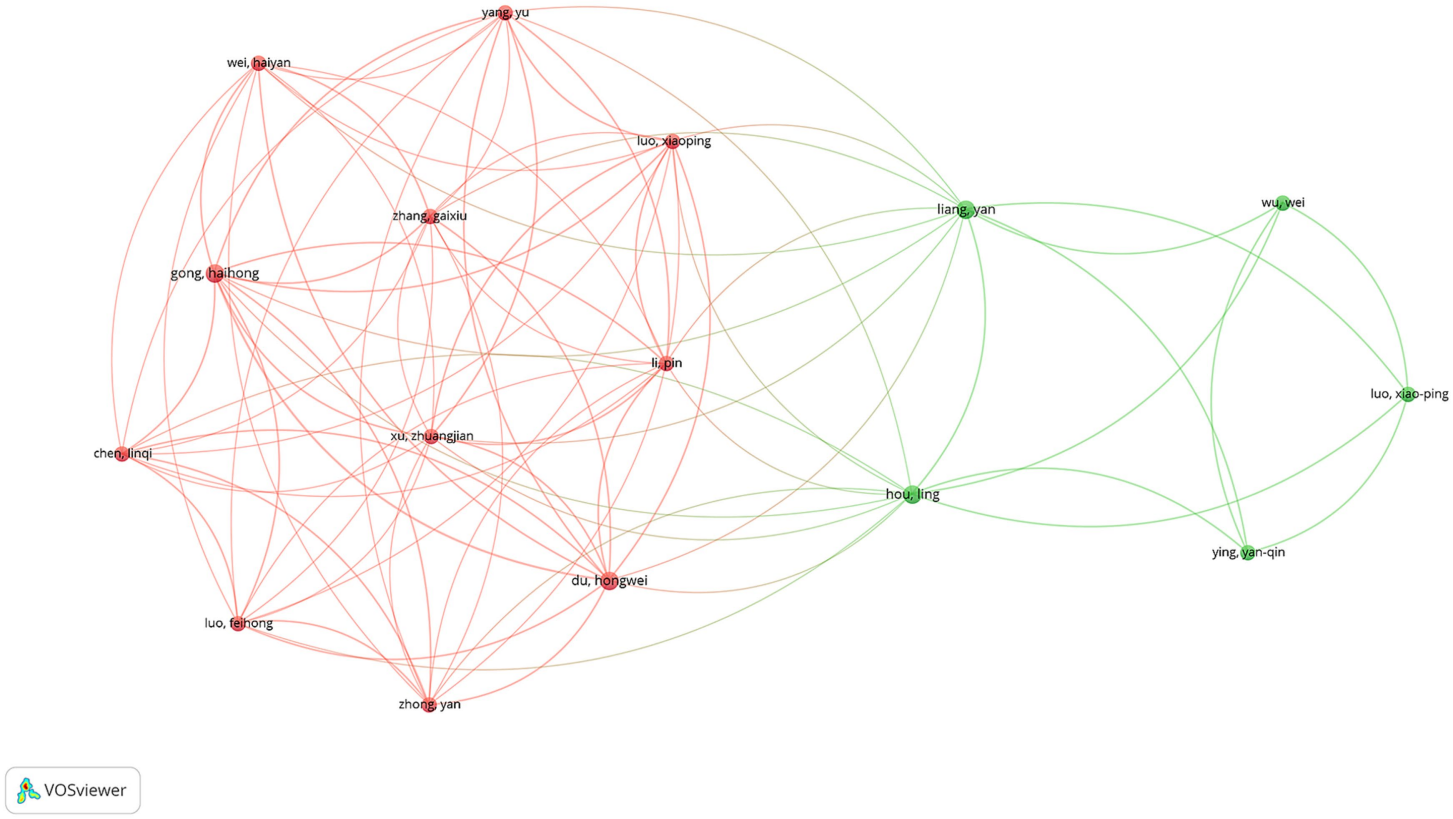
Author collaboration network in rhGH treatment in ISS.

A total of 668 unique authors contributed to the 110 articles analyzed. Focusing on international collaborations, 59 authors were identified as having contributed to at least three publications. Among these, Luo XP was the most prolific author, with 6 publications, followed by Albertsson-Wikland K and Kim HS, each with 5 publications. In terms of total citations (TCs), Albertsson-Wikland K ranked first with 171 citations, while Ranke MB was close behind with 166 citations (Supplementary Table S3). The co-authorship network analysis revealed two major clusters of collaborating authors (Figure 6). The largest cluster, highlighted in red, was centered around Yang Yu, Wei Haiyan, and Gong Haihong, indicating a strong collaborative network primarily composed of Chinese researchers. This cluster demonstrated dense interconnections, suggesting close collaboration among these authors. The second significant cluster, shown in green, was centered around Liang Yan, Wu Wei, and Hou Ling, also representing a collaborative network predominantly among Chinese researchers.

### Keyword analysis

**Figure 7:**
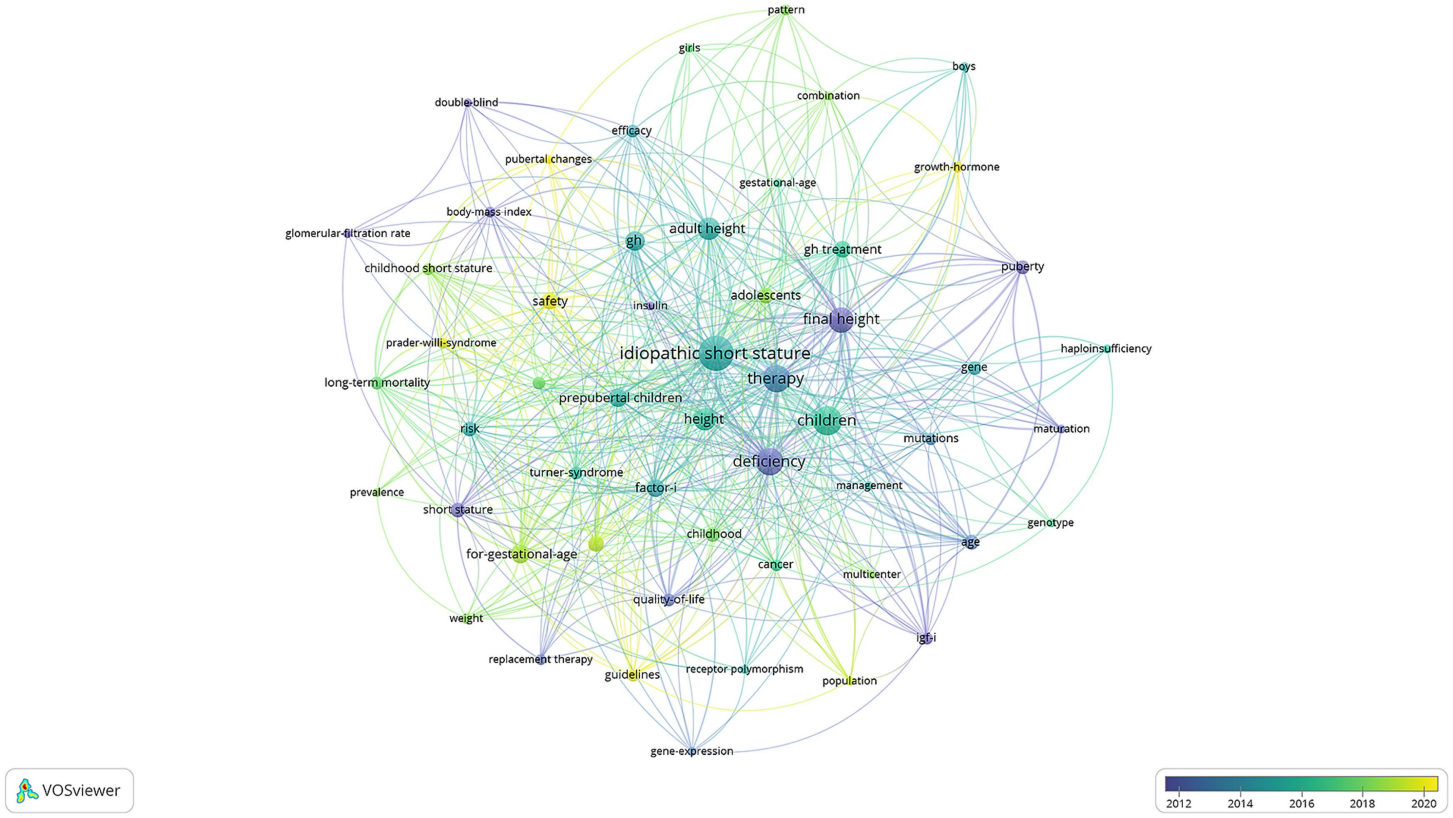
Keyword co-occurrence network in rhGH treatment in ISS.

The keyword co-occurrence analysis offers valuable insights into dominant themes and evolving research trends in rhGH therapy for ISS (Figure 7). Purple dots indicate keywords from before 2012, reflecting early research interests such as “glomerular-filtration rate” (3 occurrences, total link strength = 7), “body-mass index” (4 occurrences, total link strength = 18), “quality-of-life” (6 occurrences, total link strength = 30), and “short stature” (8 occurrences, total link strength = 30). These terms highlight the foundational role of early genetic and metabolic research, particularly in understanding growth factors. Green dots represent mature research trends around 2016, with representative keywords including “efficacy” (6 occurrences, total link strength = 23), “adult height” (17 occurrences, total link strength = 77), “long-term mortality” (6 occurrences, total link strength = 36), “risk” (8 occurrences, total link strength = 37), “for-gestational-age” (12 occurrences, total link strength = 63), and “factor I” (11 occurrences, total link strength = 44). During this period, research focused on optimizing treatment protocols, evaluating efficacy, and addressing broader clinical considerations. Yellow dots mark emerging trends from 2020 onward, with keywords such as “growth-hormone” (4 occurrences, total link strength = 11), “safety” (10 occurrences, total link strength = 30), and “pubertal changes” (3 occurrences, total link strength = 16). This recent focus emphasizes advancements in precision medicine, safety considerations, and tailoring treatments to specific age groups and developmental stages.

**Figure 8:**
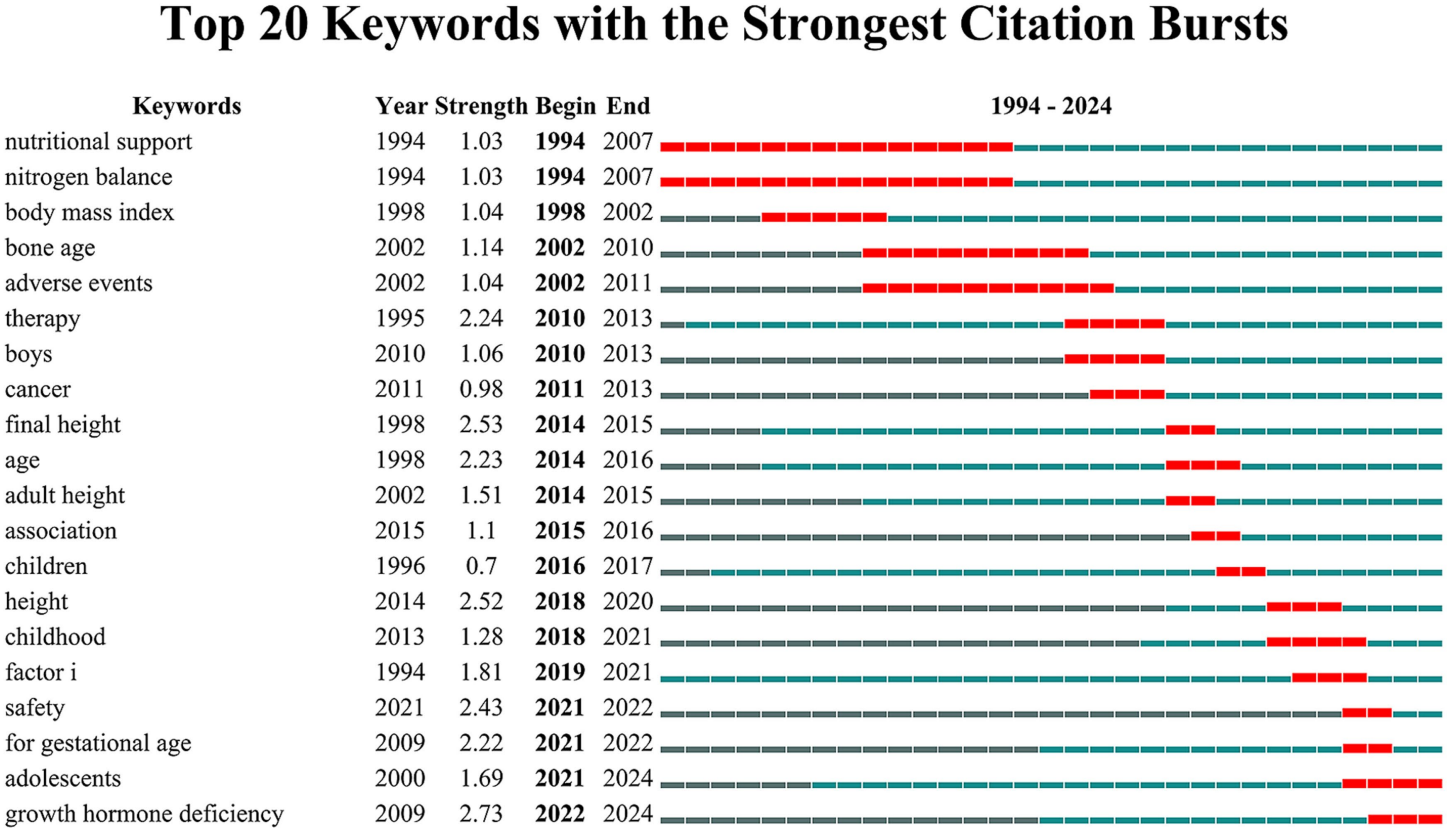
Keywords with the strongest citation bursts in rhGH treatment in ISS.

The top 20 keywords with the highest burst strengths, as shown in Figure 8, reveal shifting research focuses over time. The burst patterns at each stage align with the results from the keyword co-occurrence analysis. In the early period (1994–2010), prominent bursts included “nutritional support,” “nitrogen balance,” and “body mass index,” reflecting a focus on anthropometric measures. During the middle period (2010–2020), keywords such as “therapy,” “boys,” “cancer,” “final height,” and “adult height” highlighted research on treatment efficacy, patient subgroups, and growth outcomes. In recent years (2021–2024), the emergence of bursts for “safety,” “for gestational age,” “adolescents,” and “growth hormone deficiency” indicates heightened attention to treatment safety, specific populations, and tailored protocols for older age groups.

## Discussion

### General information

This bibliometric analysis provides a comprehensive overview of research performance on rhGH use in children with ISS, based on 33 years of data. The results reveal key trends in publication output, citation patterns, and research focus, offering a clear picture of how this field has evolved to date and what directions may emerge for future researchers. The strong upward trend in publication output—particularly the acceleration in recent years—reflects increasing interest and investment in rhGH treatment for ISS. This is consistent with the overall growth in pediatric endocrinology research and a heightened awareness of ISS as an important clinical condition (25). The peak in publications observed in 2022 may be attributed to the availability of long-term follow-up data on earlier cohorts treated with rhGH, as well as the adoption of advanced genetic and molecular techniques in ISS research (17). The field had an overall H-index of 27, suggesting a moderate to high research impact. This is comparable to values in other areas of pediatric endocrinology, such as research on growth hormone deficiency (GHD), but higher than those for rarer endocrine conditions like type 1 diabetes in children, which generally yield lower H-indices due to smaller patient populations and narrower research scope (26, 27).

A large proportion of publications appeared in specialist journals such as Journal of Clinical Endocrinology & Metabolism and Hormone Research in Paediatrics, reflecting the niche status of ISS research within the broader field. This pattern is similar to that seen in other pediatric subspecialties, where a few select journals dominate the field (28). However, the significant presence of ISS research in high-impact general endocrinology journals, especially the Journal of Clinical Endocrinology & Metabolism, highlights the broader relevance and clinical significance of rhGH treatment for ISS. Such dissemination is crucial for influencing clinical practice and policy decisions (18). The identification of key authors and institutions, notably the dominance of researchers from East Asian countries reveals an international interest for ISS research.

Our results indicate that China leads in publication output on rhGH treatment for ISS; however, most Chinese publications are single-country articles. Several factors may contribute to this pattern, as supported by recent studies. First, China’s large and diverse population, along with regional variability in the epidemiology of ISS, provides a substantial domestic patient cohort, facilitating large-scale, population-specific studies without the immediate need for international collaboration (29, 30). In addition, genetic heterogeneity and unique ISS-related mutations among Chinese children have motivated researchers to focus primarily on understanding disease mechanisms and therapeutic responses within their own demographic context (31). Second, the rapid expansion of China’s pharmaceutical industry—including the independent development and approval of domestic rhGH formulations—has fostered a research environment emphasizing local clinical trials and regulatory compliance, which further encourages national, rather than international, collaborative projects (32, 33). Third, while language barriers have historically been considered obstacles to international collaboration, recent bibliometric studies suggest that differences in regulatory frameworks, clinical practice guidelines, and healthcare infrastructure play a more significant role in limiting international co-authorship in Chinese biomedical research (34, 35). These factors collectively shape the current landscape of rhGH and ISS research in China.

These collaboration patterns have important and nuanced implications for research quality and innovation. On one hand, strong national clusters can facilitate the rapid accumulation of specialized expertise, efficient patient recruitment, and the development of context-specific treatment protocols, which is particularly relevant given the genetic and clinical heterogeneity of ISS. On the other hand, a lower rate of international collaboration (23.64%) may limit exposure to diverse methodologies, broader patient populations, and alternative research perspectives. International collaboration is often associated with higher research impact and innovation, as it brings together complementary expertise, resources, and ideas from different regions. Therefore, expanding global partnerships—especially involving countries leading in research output like China—could further enhance both the quality and generalizability of findings in this field. Nevertheless, it is important to recognize that both national focus and international cooperation have their respective advantages; a balanced approach that draws on the strengths of both models may best promote sustained progress and innovation in rhGH research for ISS.

The identification of key authors and institutions—particularly the growing dominance of researchers from East Asia—reflects the shifting global landscape of ISS research. The centralization of research within countries or single institutions can foster deep expertise, but may also risk insularity, highlighting the need for greater cross-border exchange. Enhanced international collaboration could lead to larger, more diverse patient cohorts and more robust, globally relevant conclusions, especially given variations in ISS definitions, screening, and clinical practice worldwide (36).

### Research topics and Frontiers

Over the past three decades, research on ISS and rhGH therapy has evolved from foundational studies to more nuanced investigations into safety, efficacy, and personalized treatment approaches. Temporal trends in keyword bursts demonstrate the shifting priorities of the field, corresponding to advances in both clinical practice and scientific understanding.

Early ISS research emphasized foundational concepts such as “body mass index (BMI),” “nutritional support,” “quality of life,” and “nitrogen balance”—highlighting the metabolic and physiological bases of growth disorders. Anthropometric measures like BMI served as objective indicators of growth potential and treatment efficacy, while themes such as “nutritional support” and “nitrogen balance” underscored the importance of protein metabolism and adequate nutrition for optimizing rhGH treatment outcomes (36–40).

Recent studies confirm that nutritional status continues to be a key determinant of therapeutic response, and nutritional interventions are often used in conjunction with rhGH therapy (41–43).

Over time, research focus shifted toward treatment optimization and long-term outcomes, with keywords like “efficacy,” “final height,” “adult height,” “age,” and “association” becoming central themes. This transition indicates a need to quantify treatment success using measurable endpoints and to explore the factors influencing response, such as timing, age, and baseline characteristics (44, 45). Studies evaluating variations in response by gender, age, and treatment initiation timing have supported the development of more individualized, risk-balanced treatment protocols (46, 47).

The focus on “final height” and “adult height” as benchmarks for success has led to more nuanced evaluations of rhGH efficacy, with studies showing variable gains depending on patient and treatment characteristics (48, 49). At the same time, there has been increased exploration of the psychosocial benefits of therapy, including improved self-esteem and quality of life for children with short stature (8).

In the middle phase of ISS research, keywords like “cancer” and “therapy” signaled a growing emphasis on balancing efficacy with long-term safety. While rhGH therapy advanced ISS management and addressed psychosocial concerns, concerns over potential oncological risks became a subject of scrutiny (50). Although no direct increase in cancer risk has been established for the overall ISS population, individualized risk assessment remains a key component of contemporary treatment protocols (51).

Recent years have seen the emergence of keywords such as “safety,” “pubertal changes,” “adolescents,” “precision medicine,” “growth hormone deficiency,” and “growth hormone,” reflecting a stronger focus on age-specific applications, long-term safety, and personalized medicine. The prominence of “safety” highlights ongoing concerns about potential metabolic complications, such as insulin resistance or dyslipidemia, particularly in predisposed individuals (52). This underscores the need for regular metabolic monitoring and the development of risk stratification tools.

The emergence of “pubertal changes” and “adolescents” indicates recognition of the unique challenges in treating older children, as puberty represents a critical period for growth and requires careful adjustment of dosing strategies (53). Moreover, the psychosocial and emotional dimensions of puberty call for multidisciplinary approaches that incorporate counseling and patient support (18).

The increasing presence of “precision medicine” in the literature points to advances in genetic testing and biomarker discovery that are facilitating more individualized treatments for ISS. Variants in genes such as the growth hormone receptor (GHR) and IGF-1 are now recognized as important contributors to variability in rhGH response, and artificial intelligence–driven models are beginning to support clinical decision-making in tailoring therapies (54). The appearance of “growth hormone deficiency” as a recurring keyword reflects the integration of ISS research with studies on broader growth hormone–related disorders. This trend indicates a move toward refining diagnostic criteria and optimizing therapeutic strategies for both idiopathic and deficiency-related growth impairments, supported by advances in genetic and epigenetic biomarker discovery (55).

While our bibliometric analysis primarily maps publication and citation trends, the results also reflect evolving ethical and patient-centered considerations in the literature on rhGH for ISS. The annual number of publications has shown a steady upward trajectory, particularly in recent years, paralleling a growing research focus on patient outcomes and treatment implications. Notably, our keyword analysis reveals that terms such as “quality of life” and “safety” have become increasingly prominent. Specifically, “safety” emerged in the keyword co-occurrence timeline around 2020, and its presence intensified in the burst analysis from 2021 onward, with the blue bar indicating ongoing and sustained attention to this issue. This trend highlights a shift in the field toward greater emphasis on the long-term risks and benefits of rhGH therapy, underscoring the importance of careful risk–benefit assessment in clinical decision-making. In recent years, both clinicians and families have had to weigh the potential advantages of promoting growth and psychosocial well-being against uncertainties regarding the long-term safety of rhGH use—an ethical dilemma that is increasingly recognized in the literature (56). The growing frequency and persistence of “safety” as a research focus suggest that this remains a dynamic area of ethical concern, with ongoing debate about the justification for rhGH treatment in children with non-deficiency-related short stature, and the need for individualized, informed decision-making (57).

However, despite increased attention to patient perspectives and risk assessment, explicit discussions of broader ethical debates—such as the justification for using rhGH in children without growth hormone deficiency or issues of equitable access to therapy due to cost—are still relatively underrepresented in the keyword network. This highlights a potential area for further research and scholarly discourse as the field continues to evolve (58).

## Limitations

This study has several limitations that should be considered when interpreting the findings. First, our analysis was based exclusively on the WoSCC, which may have led to the exclusion of relevant studies published in non-indexed journals or in languages other than English, introducing potential selection and language biases. Additionally, citation-based metrics, while widely used in bibliometric analyses, are subject to certain inherent limitations. For example, citation lag can result in the underrepresentation of recent publications, as newly published articles require time to accumulate citations and influence bibliometric indicators. Furthermore, the influence of self-citations was not specifically addressed in this study, which could potentially inflate the apparent impact of certain authors or institutions. Finally, given the dynamic and evolving nature of scientific research, our analysis may not fully capture the impact of the latest studies or adequately reflect emerging topics, thus potentially underestimating recent trends within the field.

## Conclusion

This bibliometric analysis highlights significant progress in rhGH treatment for ISS over the past three decades. Research has expanded globally, with major contributions from China, the United States, and South Korea, and leading institutions such as the University of Ulsan and Shandong University. Early studies focused on physiological mechanisms, safety, and metabolic factors, while recent efforts emphasize precision medicine, age-specific applications, and long-term outcomes. Future priorities include addressing the long-term impacts of rhGH therapy and ensuring equitable, ethical implementation of advanced treatments.

## Data Availability

The original contributions presented in the study are included in the article/Supplementary material, further inquiries can be directed to the corresponding author.
