# Supplementary material for: Global trends in recombinant human growth hormone for the treatment of idiopathic short stature: a bibliometric analysis
Source: Front Med (Lausanne). 2025 Aug 6;12:1577396. doi: 10.3389/fmed.2025.1577396 (PMC12364896; doi:10.3389/fmed.2025.1577396)
Supplement: Supplementary file 1 [file Supplementary_file_1.docx]

**Table S1 Bibliometric Indicators of High-Impact Journals**

| **Journal** | **H_index** | **IF** | **JCR_Quartile** | **PY_start** | **TP** | **TP_rank** | **TC** | **TC_rank** |
| --- | --- | --- | --- | --- | --- | --- | --- | --- |
| HORMONE RESEARCH IN PAEDIATRICS | 7 | 2.6 | 1 | 2011 | 11 | 1 | 144 | 2 |
| JOURNAL OF PEDIATRIC ENDOCRINOLOGY & METABOLISM | 7 | 1.3 | 3 | 1998 | 10 | 2 | 82 | 7 |
| JOURNAL OF CLINICAL ENDOCRINOLOGY & METABOLISM | 6 | 5.0 | 1 | 2000 | 8 | 3 | 623 | 1 |
| CLINICAL ENDOCRINOLOGY | 3 | 3.0 | 2 | 1993 | 3 | 7 | 115 | 5 |
| GROWTH HORMONE & IGF RESEARCH | 3 | 1.6 | 4 | 2015 | 3 | 8 | 72 | 9 |
| PLOS ONE | 3 | 2.9 | 1 | 2015 | 4 | 6 | 32 | 15 |
| ACTA PAEDIATRICA | 2 | 2.4 | 1 | 1993 | 2 | 9 | 37 | 11 |
| ADVANCES IN THERAPY | 2 | 3.4 | 2 | 2008 | 2 | 10 | 3 | 140 |
| ENDOCRINE | 2 | 3.0 | 2 | 2000 | 4 | 4 | 10 | 50 |
| EUROPEAN JOURNAL OF ENDOCRINOLOGY | 2 | 5.3 | 1 | 2016 | 2 | 11 | 91 | 6 |
| EXPERIMENTAL AND THERAPEUTIC MEDICINE | 2 | 2.4 | 3 | 2016 | 2 | 12 | 3 | 151 |
| FRONTIERS IN ENDOCRINOLOGY | 2 | 3.9 | 2 | 2018 | 4 | 5 | 13 | 34 |
| HORMONE AND METABOLIC RESEARCH | 2 | 2.0 | 3 | 1998 | 2 | 13 | 6 | 77 |
| HORMONE RESEARCH | 2 | 0 | - | 2000 | 2 | 14 | 138 | 3 |
| JOURNAL OF TRAUMA-INJURY INFECTION AND CRITICAL CARE | 2 | 0 | - | 1991 | 2 | 16 | 6 | 80 |
| PEDIATRICS | 2 | 6.2 | 1 | 2010 | 2 | 18 | 49 | 10 |
| AMERICAN JOURNAL OF MANAGED CARE | 1 | 2.5 | 1 | 2013 | 1 | 19 | 1 | 310 |
| ANALYTICAL CHEMISTRY | 1 | 6.7 | 1 | 2017 | 1 | 21 | 4 | 103 |
| ANGLE ORTHODONTIST | 1 | 3.0 | 1 | 1995 | 1 | 22 | 1 | 318 |
| BEST PRACTICE & RESEARCH CLINICAL ENDOCRINOLOGY & METABOLISM | 1 | 6.1 | 1 | 2015 | 1 | 25 | 15 | 30 |

Note(s): H_index: The h-index of the journal, which measures both the productivity and citation impact of the publications. IF: Impact Factor, indicating the average number of citations to recent articles published in the journal. JCR_Quartile: The quartile ranking of the journal in the Journal Citation Reports, indicating the journal's ranking relative to others in the same field (Q1: top 25%, Q2: 25%-50%, Q3: 50%-75%, Q4: bottom 25%). TP: Total Publications. TP_rank: Rank of Total Publications. TC: Total Citations. TC_rank: Rank of Total Citations. Average Citations: The average number of citations per publication. PY_start: Publication Year Start, indicating the year the journal started publication.

**Table S2 Publication and Citation Profiles of Leading Countries**

| **Country** | **Articles** | **Freq** | **SCP** | **MCP** | **MCP_Ratio** | **TP** | **TP_rank** | **TC** | **TC_rank** | **Average Citations** |
| --- | --- | --- | --- | --- | --- | --- | --- | --- | --- | --- |
| CHINA | 36 | 0.327 | 35 | 1 | 0.028 | 145 | 1 | 141 | 4 | 3.9 |
| USA | 24 | 0.218 | 18 | 6 | 0.250 | 70 | 2 | 481 | 1 | 20 |
| SOUTH KOREA | 9 | 0.082 | 9 | 0 | 0.000 | 58 | 3 | 111 | 5 | 12.3 |
| SWEDEN | 7 | 0.064 | 6 | 1 | 0.143 | 31 | 4 | 171 | 3 | 24.4 |
| GERMANY | 5 | 0.045 | 3 | 2 | 0.400 | 16 | 9 | 220 | 2 | 44 |
| NETHERLANDS | 5 | 0.045 | 4 | 1 | 0.200 | 19 | 6 | 109 | 6 | 21.8 |
| BRAZIL | 4 | 0.036 | 3 | 1 | 0.250 | 12 | 10 | 55 | 7 | 13.8 |
| UNITED KINGDOM | 4 | 0.036 | 3 | 1 | 0.250 | 17 | 7 | 33 | 9 | 8.2 |
| ITALY | 3 | 0.027 | 3 | 0 | 0.000 | 22 | 5 | 10 | 15 | 3.3 |
| FRANCE | 2 | 0.018 | 2 | 0 | 0.000 | 16 | 8 | 5 | 19 | 2.5 |
| ARGENTINA | 1 | 0.009 | 1 | 0 | 0.000 | 1 | 22 | 12 | 11 | 12 |
| AUSTRIA | 1 | 0.009 | 1 | 0 | 0.000 | 2 | 20 | 50 | 8 | 50 |
| BELGIUM | 1 | 0.009 | 0 | 1 | 1.000 | 8 | 12 | 5 | 18 | 5 |
| CANADA | 1 | 0.009 | 0 | 1 | 1.000 | 5 | 15 | 1 | 20 | 1 |
| COSTA RICA | 1 | 0.009 | 0 | 1 | 1.000 | 1 | 23 | 13 | 10 | 13 |
| CYPRUS | 1 | 0.009 | 1 | 0 | 0.000 | 5 | 16 | 12 | 12 | 12 |
| DENMARK | 1 | 0.009 | 0 | 1 | 1.000 | 6 | 13 | 10 | 14 | 10 |
| NEW ZEALAND | 1 | 0.009 | 0 | 1 | 1.000 | 4 | 17 | 6 | 16 | 6 |
| QATAR | 1 | 0.009 | 0 | 1 | 1.000 | 3 | 19 | 6 | 17 | 6 |
| SWITZERLAND | 1 | 0.009 | 1 | 0 | 0.000 | 9 | 11 | 12 | 13 | 12 |

Note(s): Articles: Publications of Corresponding Authors only. Freq: Frequence of Total Publications. MCP_Ratio: Proportion of Multiple Country Publications. TP: Total Publications. TP_rank: Rank of Total Publications. TC: Total Citations. TC_rank: Rank of Total Citations. Average Citations: The average number of citations per publication.

**Table S3 Publication and Citation Profiles of High-Impact Authors**

| **Authors** | **h_index** | **g-index** | **m-index** | **PY_start** | **TP** | **TP_Frac** | **TP_rank** | **TC** | **TC_rank** |
| --- | --- | --- | --- | --- | --- | --- | --- | --- | --- |
| KIM HS | 5 | 5 | 0.455 | 2014 | 5 | 0.51 | 3 | 86 | 7 |
| ALBERTSSON-WIKLAND K | 4 | 5 | 0.444 | 2016 | 5 | 0.58 | 2 | 171 | 1 |
| LEE KH | 4 | 4 | 0.364 | 2014 | 4 | 0.37 | 5 | 45 | 10 |
| YOO HW | 4 | 4 | 0.333 | 2013 | 4 | 0.37 | 7 | 45 | 10 |
| CHEN LQ | 3 | 3 | 1.000 | 2022 | 3 | 0.16 | 8 | 9 | 24 |
| CHUNG WY | 3 | 3 | 0.273 | 2014 | 3 | 0.27 | 9 | 38 | 14 |
| CLAYTON P | 3 | 3 | 0.333 | 2016 | 3 | 0.38 | 10 | 72 | 8 |
| GEFFNER ME | 3 | 3 | 0.273 | 2014 | 3 | 0.39 | 12 | 35 | 19 |
| HOU L | 3 | 3 | 0.429 | 2018 | 3 | 0.41 | 14 | 26 | 22 |
| HWANG JS | 3 | 3 | 0.273 | 2014 | 3 | 0.27 | 15 | 38 | 14 |
| JIN DK | 3 | 3 | 0.429 | 2018 | 3 | 0.28 | 16 | 29 | 21 |
| KO CW | 3 | 3 | 0.273 | 2014 | 3 | 0.27 | 17 | 38 | 14 |
| LUO XP | 3 | 5 | 0.300 | 2015 | 6 | 0.67 | 1 | 35 | 19 |
| MASSA GG | 3 | 3 | 0.111 | 1998 | 3 | 0.48 | 19 | 97 | 5 |
| MILLER BS | 3 | 3 | 0.273 | 2014 | 3 | 0.53 | 20 | 39 | 13 |
| RANKE MB | 3 | 3 | 0.094 | 1993 | 3 | 0.63 | 21 | 166 | 2 |
| REKERS-MOMBARG LTM | 3 | 3 | 0.111 | 1998 | 3 | 0.48 | 22 | 97 | 5 |
| ROGOL AD | 3 | 3 | 0.176 | 2008 | 3 | 0.82 | 23 | 26 | 22 |
| SANDBERG DE | 3 | 3 | 0.120 | 2000 | 3 | 1.33 | 24 | 54 | 9 |
| SHIN CH | 3 | 3 | 0.250 | 2013 | 3 | 0.28 | 25 | 44 | 12 |

Note(s): H_index: The h-index of the journal, which measures both the productivity and citation impact of the publications. g_index: The g-index of the journal, which gives more weight to highly-cited articles. m_index: The m-index of the journal, which is the h-index divided by the number of years since the first published paper. TP: Total Publications. TP_rank: Rank of Total Publications. TC: Total Citations. TC_rank: Rank of Total Citations. Average Citations: The average number of citations per publication. PY_start: Publication Year Start, indicating the year the journal started publication.
