# Supplementary material for: Global trends in recombinant human growth hormone for the treatment of idiopathic short stature: a bibliometric analysis
Source: Front Med (Lausanne). 2025 Aug 6;12:1577396. doi: 10.3389/fmed.2025.1577396 (PMC12364896; doi:10.3389/fmed.2025.1577396)
Supplement: Supplementary file 2 [file Supplementary_file_2.docx]

**Table S4. Overview of Statistical Techniques Used and Their Applications in Figures.**

| Statistical methods | Explanation | Application |
| --- | --- | --- |
| VOSviewer (version 1.6.20) | VOSviewer is a widely used bibliometric analysis software that allows retrieving essential information from numerous publications. This is heavily used to build collaboration, co-citation and co-occurrence networks. This map represents various projects through the nodes. The size and colour of the node indicate how many items exist within as well as which type they are. The weight of the lines connecting nodes indicates how much those projects have collaborated or co-cited. | Figure 2. Trends in Publications in rhGH treatment in ISS from 1991 to 2024. B. Detailed trend analysis showing the annual fluctuations in publication volume.  Figure 3. Network Analysis of Journals in rhGH treatment in ISS. A. Co-occurrence Networks of Journals, reflecting thematic or topical connections based on co-citations. B. Coupling Networks of Journals, illustrating shared intellectual foundations through common references.  Figure 4. Global Distribution and Collaboration in rhGH treatment in ISS. B. V Collaboration Map among Countries.  Figure 5. Institutional Contributions and Collaborations in rhGH treatment in ISS. B. Collaboration Map among Institutions.  Figure 6. Author Collaboration Network in rhGH treatment in ISS.  Figure 7. Keyword Co-occurrence Network in rhGH treatment in ISS. |
| CiteSpace (version 6.3. R1) | CiteSpace was used to detect keyword emergence and burst. H index was employed to quantify the academic impact of individuals and journals, which is a vital indication to evaluate the academic contribution of researchers and could predict their future scientific achievements (1, 2) | Figure 8. Keywords with the Strongest Citation Bursts in rhGH treatment in ISS. |
| R package “Bibliometrix” (version4.3.3) | R was utilized to export descriptive analysis results, a process also known as performance analysis within the bibliometric domain (3) | Figure 2. Trends in Publications in rhGH treatment in ISS from 1991 to 2024. A. Graphical overview of the total publications per year.  Figure 4. Global Distribution and Collaboration in rhGH treatment in ISS. A. Corresponding Authors' Publications by Country, differentiating between Single Country Publications (SCP) and Multiple Country Publications (MCP).  Figure 5. Institutional Contributions and Collaborations in rhGH treatment in ISS. A. Top 10 Institutions by Article Count and Ran. |

1. Bertoli-Barsotti L, Lando T. A theoretical model of the relationship between the h-index and other simple citation indicators. Scientometrics. 2017;111(3):1415-48.

2. Hirsch JE. An index to quantify an individual's scientific research output. Proc Natl Acad Sci U S A. 2005;102(46):16569-72.

3. Donthu N, Kumar S, Mukherjee D, Pandey N, Lim WM. How to conduct a bibliometric analysis: An overview and guidelines. Journal of Business Research. 2021;133:285-96.
